# Therapeutic Anti-Depressant Potential of Microbial GABA Produced by *Lactobacillus rhamnosus* Strains for GABAergic Signaling Restoration and Inhibition of Addiction-Induced HPA Axis Hyperactivity

**DOI:** 10.3390/cimb44040096

**Published:** 2022-03-22

**Authors:** Fernanda-Marie Tette, Samuel K. Kwofie, Michael D. Wilson

**Affiliations:** 1 Department of Biomedical Engineering, School of Engineering Sciences, College of Basic and Applied Sciences, University of Ghana, Legon, Accra LG 77, Ghana; fmtette@gmail.com; 2 West African Centre for Cell Biology and Infectious Pathogens, Department of Biochemistry, Cell and Molecular Biology, University of Ghana, Legon, Accra LG 54, Ghana; 3 Department of Parasitology, Noguchi Memorial Institute for Medical Research (NMIMR), College of Health Sciences (CHS), University of Ghana, Legon, Accra LG 581, Ghana; mwilson@noguchi.ug.edu.gh; 4 Department of Medicine, Loyola University Medical Center, Maywood, IL 60153, USA

**Keywords:** addiction, corticotropin-releasing factor (CRF), depression, gamma-aminobutyric acid (GABA), gut microbiome, hypothalamic–pituitary–adrenal (HPA) axis, *Lactobacillus rhamnosus*, probiotics

## Abstract

The role of the microbiota–gut–brain (MGB) axis in mood regulation and depression treatment has gained attention in recent years, as evidenced by the growing number of animal and human studies that have reported the anti-depressive and associated gamma-aminobutyric acid-ergic (GABAergic) effects of probiotics developed from *Lactobacillus rhamnosus* bacterial strains in the gut microbiome. The depressive states attenuated by these probiotics in patients suffering from clinical depression also characterize the severe and relapse-inducing withdrawal phase of the addiction cycle, which has been found to arise from the intoxication-enabled hyperregulation of the hypothalamic–pituitary–adrenal (HPA) axis, the body’s major stress response system, and a corresponding attenuation of its main inhibitory system, the gamma-aminobutyric acid (GABA) signaling system. Therefore, the use of probiotics in the treatment of general cases of depression provides hope for a novel therapeutic approach to withdrawal depression remediation. This review discusses potential therapeutic avenues by which probiotic application of *Lactobacillus rhamnosus* strains can be used to restore the central GABAergic activity responsible for attenuating the depression-inducing HPA axis hyperactivity in addiction withdrawal. Also, information is provided on brain GABAergic signaling from other known GABA-producing strains of gut microbiota.

## 1. Introduction

According to the 2021 World Drug Report from the United Nations Office on Drugs and Crime (UNODC), over 36 million people globally suffer from the substance use disorder commonly known as addiction [1]. This condition is a chronically relapsing disorder involving compulsive drug-seeking and ingestion, poor self-control in limiting drug intake, and the onset of negative emotional states when prevented from using drugs. This condition is characterized by a three-stage recurring cycle comprising intoxication, withdrawal and anticipation [2]; withdrawal, the second phase of this cycle, is characterized by depressive or negative emotional states that include chronic irritability, emotional pain, malaise and loss of motivation for natural rewards [2,3]. These maladaptive states can be traced back to the dysregulated hyperactivity of the body’s primary stress regulation system, the hypothalamic–pituitary–adrenal (HPA) axis, comprising the hypothalamus, pituitary, and adrenal glands [4].

**Figure 1:**
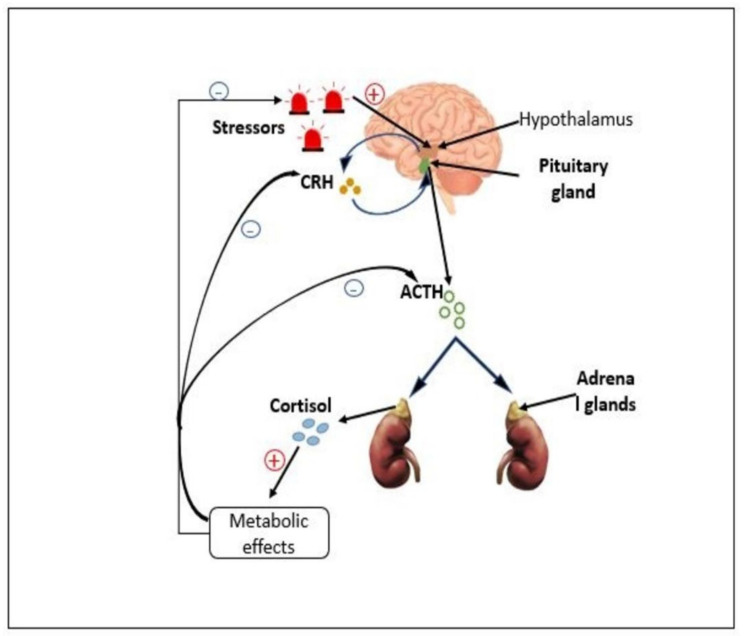
The hypothalamic–pituitary–adrenal (HPA) axis. As a stress response, corticotropin-releasing factor (CRF) is produced within the hypothalamus and then released into the anterior pituitary gland, where it then induces the secretion of adrenocorticotropic hormone (ACTH) into the systemic circulation. The ACTH then stimulates cortisol synthesis in the adrenal cortex, which is then released into the systemic circulation as a positive feedback mechanism for initiating necessary metabolic effects for stressor adaptation. After peak cortisol levels, negative feedback is initiated in the hypothalamus and anterior pituitary, inhibiting CRF and ACTH production, respectively, and ultimately ceasing the stress response.

Under normal conditions within the HPA axis, corticotropin-releasing factor (CRF), also known as corticotropin-releasing hormone (CRH), is produced within the central nervous system (CNS) (Figure 1) by neurons in the paraventricular nucleus (PVN) of the hypothalamus in response to stressors, and it is released into the hypophysial portal vessels that access the anterior pituitary gland [5]. This CRF then binds to the CNS, predominantly with the CRF receptor, corticotropin-releasing factor receptor 1 (CRFR1) on pituitary corticotropes, inducing the further release of adrenocorticotropic hormone (ACTH) from endocrine cells into the systemic circulation. ACTH then stimulates cortisol synthesis in the adrenal cortex, which is then released into the systemic circulation to regulate adaptive physiological changes [6]. Next, after cortisol synthesis and secretion, inhibitory mechanisms take place in the CRF signaling pathway to decrease HPA axis activity in order to initiate stress recovery. The primary inhibitory mechanism is gamma-aminobutyric acid-ergic (GABAergic) signaling [7] initiated in a negative feedback microcircuit whereby cortisol (after peak levels) binds to glucocorticoid receptors (GCs) and mineralocorticoid receptors (MRs), inhibiting glutamate release and reducing the excitation of CRF neurons [8]. GABA receptor expression on CRF neurons is also increased as glutamatergic activity decreases, enabling PVN CRFR1 neurons to create recurrent GABAergic synaptic connections with active CRF neurons [9]. The GABAergic signaling across synapses thus inhibits CRF neuronal excitability and consequently attenuates HPA axis activity, prevents axis dysregulation, and maintains homeostasis [9,10].

During withdrawal, however, the HPA axis is hyperactivated through the prior chronic overactivity of the dopaminergic system initiated during the preceding intoxication phase of the addiction cycle [2]. This results in a steep rise in CRF synthesis and a hyper-activity of the CRF receptor signaling pathway in the extended amygdala and the ventral tegmental area, causing a steep decline in dopamine release and a corresponding spike in cortisol production, with neuroinhibitory GABAergic signaling also being attenuated during withdrawal as a cascading effect of the hypersecretion of the glucocorticoid, cortisol [2,6].

Because cortisol is a prime stress hormone, its increased secretion into the systemic circulation generates highly elevated levels of stress, with depressive states manifesting as major symptoms and persisting until therapeutic interventions are performed to upregulate the GABAergic activity in the HPA axis [2]. Such interventions have been discovered in the field of probiotics using commensal bacteria present in the gastrointestinal tract capable of regulating neurobiological systems in the CNS via the microbiota–gut–brain axis [10,11]. Among these systems is the CNS GABAergic signaling system, and a key bacterium found to have regulatory effects on this system is *Lactobacillus rhamnosus*, regarding which studies have reported findings such as increases in CNS GABAergic activity and corresponding reductions in depression-associated behavior in both mice and humans upon the ingestion of its bacterial strains [12].

This review aims to discuss the processes by which *Lactobacillus rhamnosus* strains regulate and boost CNS GABAergic signaling as potential therapeutic avenues by which such strains can be used to restore the central GABAergic activity responsible for eliminating the depression-inducing HPA axis hyperactivity in addiction withdrawal. The discourse is additionally enriched with snapshots on other known GABA-producing strains of gut microbiota and their contributions to brain GABAergic signaling.

## 2. GABA and GABA Receptors

γ-aminobutyric acid (GABA) is a naturally occurring amino acid found in plants, microorganisms and animals. It is the primary neurotransmitter responsible for neuroinhibition in the central nervous system (CNS), i.e., the process of reducing neuronal excitability [13]. GABA is critical in the regulation of the responsiveness, excitability and synchronization of cortical neuronal signaling, so it regulates factors such as emotional states, cognition and memory, circadian rhythms, and neural development [14]. Accordingly, GABA signaling dysfunction has been implicated as a causative factor in many neurologic and psychiatric conditions, and GABA signaling modulation has been foundational in multiple pharmacologic therapeutic developments in psychiatry and neurology [15,16].

**Figure 2:**
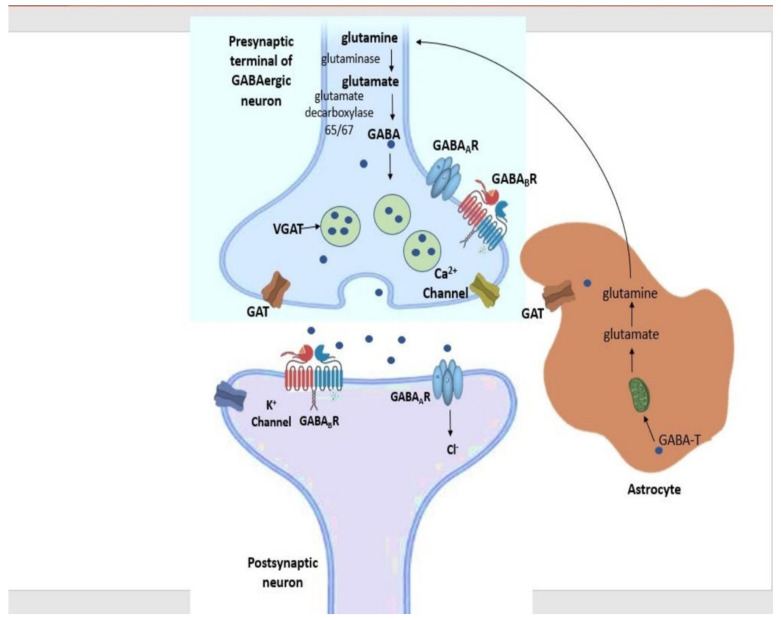
GABAergic Signaling. Glutamine is first converted to glutamate via action of the enzyme glutaminase. GABA is synthesized from the breakdown of glutamate by glutamic acid decarboxylase (GAD). GABA is then transported to the synaptic vesicle of the pre-synaptic terminal through the vesicular GABA transporter (vGAT) and is released into the synapse, where it binds to either of the GABA receptors GABA_A_ or GABA_B_ on the postsynaptic neuron, thus inhibiting it. Upon inhibition, excess GABA is moved from the synapse and into astrocytes via GABA transporters (GATs), where it is ultimately converted back to glutamate and then glutamine through the initiatory action of GABA transaminase (GABA-T) within the mitochondria.

GABA synthesis (Figure 2) is initiated in the cytoplasm of the pre-synaptic GABAergic neurons by the breakdown of astrocyte-produced glutamine to glutamate via the action of the enzyme glutaminase [17]. Then, glutamate undergoes alpha-decarboxylation via the enzyme glutamic acid decarboxylase (GAD). GABA is then transferred into synaptic vesicles by the vesicular GABA transporter (vGAT) and, upon membrane depolarization, is released into the synaptic cleft [14].

When released, GABA can bind to any of its two major receptors on the post-synaptic neuron: GABA type A (GABA_A_) receptors or GABA type B (GABA_B_) receptors. GABA_A_ receptors are ionotropic receptors mediating fast inhibitory signals through rapid postsynaptic membrane hyperpolarization, and GABA_B_ receptors are metabotropic G-protein coupled receptor producing slow and prolonged inhibitory signals on the postsynaptic membrane, resulting in post-synaptic neuron inhibition [18,19]. Alternatively, GABA is moved into synaptic vesicles and then transferred back into the presynaptic neuron or moved into neighboring astrocytes, where it is metabolized by mitochondrial GABA transaminase (GABA-T) into succinate semialdehyde (SSA) and then ultimately converted back to glutamine for neuronal uptake [14].

In recent studies in probiotics, GABA has been reported to be produced by bacteria in the gut microbiome in a similar manner as synthesized in the CNS, especially by bacteria belonging to the *Lactobacillus* genera [20]. Furthermore, some experimental strains of the *Lactobacillus* genera have been reported to initiate GABAergic signaling in mice across the microbiota–gut–brain (MGB) axis and directly into the PVN neurons responsible for initiating HPA axis activity, generating anti-depressant effects. These strains include *Lactobacillus rhamnosus* JB-1 [20,21].

### GABA-Ergic Signaling across the Microbiota–Gut–Brain (MGB) Axis

**Figure 3:**
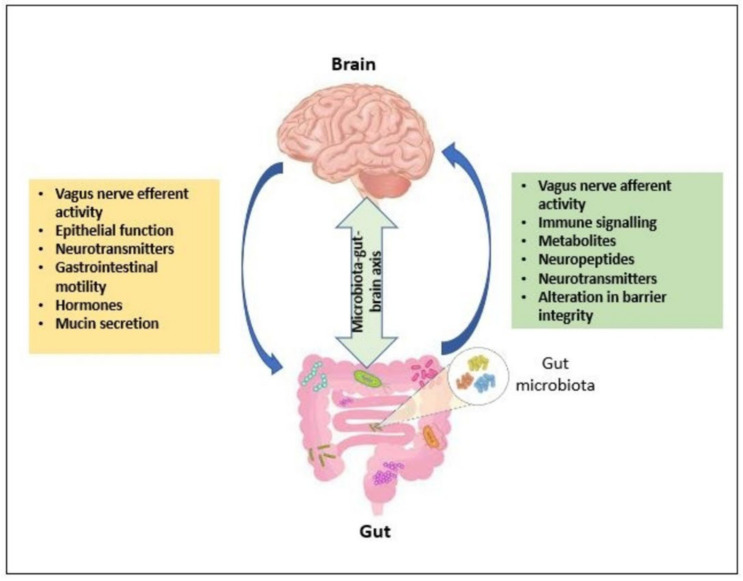
The microbiota–gut–brain axis. The bi-directional channel of communication between the brain, gut, and microbiota occurs in an upstream and downstream process. Upstream signaling mechanisms such as vagal afferent signaling and immune signaling are initiated from the gut through synthesis of metabolites, neuropeptides, and neurotransmitters by the gut microbiota. This influences the central nervous system (CNS) activity in response to factors within the gut environment such as alteration in intestinal barrier integrity. Downstream neurotransmitter and hormone activity within the CNS can initiate vagal efferent signaling and other metabolic pathways to influence gut environment properties such as gastrointestinal motility, mucin secretion, and epithelial function.

The microbiota–gut–brain (MGB) axis (Figure 3) is the bi-directional network between the gut microbiome and the brain involving endocrine, immune, and neurotransmitter systems, with the vagus nerve (VN) as its communication interface [12]. The gut microbiome is the totality of all commensal and pathogenic microorganisms including bacteria, viruses, protozoa, and fungi (with approximately 10^14^ species) and their collective genetic material present in the gastrointestinal tract [10]. In the gut microbiome, many microbes are commensal and promote health such that they can be ingested to improve host physiology. Upon ingestion, some of these intestinal microbes regulate anxiety and depression-like behavior, as well as modulate GABA-ergic, glutaminergic NMDA, and serotonergic 5HT1A receptors in the brain [10,12]. These ingested microbes, commonly belonging to the *Lactobacilli* and *Bifidobacteria* genera, are termed probiotics [11]. These probiotics are normally ingested in the form of capsules, powder, or fermented products. The probiotics have been used in MGB axis modulation by releasing molecules including metabolites, such as short chain fatty acids and liposaccharides, and neurotransmitters, such as gamma-aminobutyric acid (GABA), dopamine, and serotonin. These act locally on the enteric nervous system, where some access the CNS through either circumventricular organs or the vagus nerve, and some have been reported to be effective in reducing clinical depression [11,21,22,23,24].

The vagus nerve, one of the major pathways for CNS access across the MGB axis, is the longest cranial nerve of the nervous system and links the central nervous system to the body by the innervation of the lungs, gastrointestinal tract, and the heart [25]. This structure comprises 80% afferent fibers responsible for signal transmission from the viscera to the CNS and 20% efferent fibers responsible for signal transmission from the CNS to the viscera [26]. When the vagus nerve senses the microbiota-secreted neuroactive compounds and metabolites through its afferent fibers, it initiates neural activation in the nucleus of the solitary tract (NTS) of the CNS—the first entrance of vagal afferents in the brain. This sensory gut information transferred to the NTS is then integrated into its widespread projections such as the paraventricular nucleus of the hypothalamus (PVH) and the amygdala, which are parts of the central autonomic network (CAN) [26]. This information is then used within the CAN to generate an adaptive response, such as the activation of GABAergic signaling to inhibit CRF-producing neurons in the paraventricular nucleus [12].

Through harnessing the use of the microbiota–gut–brain axis, various studies have thus shown the efficacy of possible treatments that explicitly target the use of the gut–brain axis [22,27,28,29,30,31,32,33].

In studies on emotional behavior regulation, gut vagal afferents have been found to be modulators of anxiety and learned fear in rat models of subdiaphragmatic vagal deafferentation [28]. With the rats’ abdominal vagal afferents severed, with only half of efferents preserved, low levels of anxiety-like behavior were observed with elevated levels of auditory-induced fear, implicating vagal afferent signaling as a regulator of anxiety-related behavior [28]. The authors of another study investigated the impact of gut microbiota modification on metabolic abnormalities and depression-related behavior in both obesity and diabetes. Insulin action and behavior in diet-induced obesity (DIO) mice were analyzed in the presence and absence of the antibiotics, metronidazole and vancomycin [30]. Due to the anti-bacterial annihilation of many bacterial classes, with the main exception of Firmicutes of the Bacillus class, the effects associated with this overhaul of gut microbial composition were the enhancement of CNS insulin signaling and reduced anxiety and depression correlating with alterations in amino acids, GABA, brain-derived neurotropic factor (BDNF), tryptophan, and acylcarnitines [30].

In studies on epilepsy, the gut microbiota were reported to regulate anti-seizure effects [27]. In an investigation on microbial involvement in ketogenic diet anti-seizure effects carried out on two mouse models of intractable epilepsy, results showed that through the administration of KD-associated microbiota of the *Akkermansia* and *Parabacteroides* genera, protection against acute and spontaneous seizures was assured, with correlations of both elevated GABA levels, resulting in an increase in hippocampal GABA/glutamate ratio and a subsequent inhibition of gamma-glutamylation contributing to seizure protection of the hamster models [27].

The effects of gut microbial profile alteration in autism spectrum disorder (ASD) in children aged 2–13 years [29] were also demonstrated. The effects of ASD on gastrointestinal symptoms such as chronic constipation were investigated through metagenomics analysis revealing the absence of species diversity, and liquid chromatography mass spectrometry showing a corresponding metabolic dysregulation compared to the respective gut microbial and metabolite profiles of typically developing (TD) children [29]. The bacterial species absent were those of the *Prevotella, Bacteroides,* and *Sutterella* genera. The corresponding abnormality in neurotransmitter levels such as GABA, serotonin, and histidine were due to the microbial-associated alteration in levels of metabolites belonging to their metabolic network [29]. The authors of a study on autism who investigated the therapeutic strength of probiotics genera *Bifidobacteria* and *Lactobacilli* commonly found in the gastrointestinal tract, reported that bacterial strains of these genera in autistic models of hamsters could correct autistic-like excitation/inhibition imbalances in the CNS by increasing GABA levels and consequently reducing glutamate concentrations responsible for toxic neuroexcitation in subjects [30].

Furthermore, recent studies in probiotics have demonstrated that through the vagus nerve, some gut microbiota have been found to increase the CNS level of GABA in mice, modulating GABA_A_ and GABA_B_ receptor expression by synthesizing GABA and activating GABA signaling pathways via vagal afferents [31,32]. It has also been shown that the GABA produced in the gut can cross the intestinal barrier through the proton-coupled amino acid transporter, hPAT1, therefore allowing microbial GABA to interact with GABA receptors and transporters widespread on vagus afferents, as well as widespread NTS projections such as the paraventricular nucleus (PVN) of the hypothalamus, to effectively lower HPA axis hyperactivity [10,33].

## 3. Probiotics

Probiotics, according to the World Health Organization, are “live microorganisms that, when administered in adequate amounts, confer a health benefit on the host” [34]. These organisms are generally sourced from human subjects, with potent resistance to harsh intestinal conditions such as high acidity, enzyme actions, and biliary salts [35]. They are also devoid of toxicity factors and are antagonistic to pathogens because they alter the binding of the toxins produced by them, in effect deactivating them and enabling gut detoxification. Many also play large roles in immunomodulation and also maintain microbial balance in the host [36,37].

Many of these characteristics contribute to the health benefits they provide to their hosts, which include the prevention and treatment of intestinal diseases, such as infectious diarrhea and antibiotic-associated diarrhea, inflammatory bowel disease (IBD), irritable bowel syndrome (IBS), lactose intolerance, and allergies in children [38,39], as well as psychiatric ailments such as major depressive disorder [10]. Probiotics are thus classified based on different functional varieties such as psychobiotics, which are bacteria ingested to deliver mental health benefits, and immunobiotics, which are microbes whose prime benefit is host immunomodulation [36,37,40].

It is also important to note that despite the widely proven benefits of probiotics and their generally minimal side effects, the disadvantage of their use has been significantly observed in higher risk groups, including the elderly, pregnant women, immunocompromised patients, chronically ill individuals, and patients suffering from malnutrition, as well as infants, preterm babies and neonates still in the development phase of the immune system [41]. These groups normally experience infections and ailments such as endocarditis, sepsis, and invasive fungemia [42,43,44].

However, among the general population not at risk, many of these microorganisms are ingested in the form of dietary supplements, natural herbal remedies, and pharmaceutical drugs [45]. Many of these ingested organisms are species of bacterial genera such as those outlined in Table 1, which are among the core genera commonly used in probiotics applications. They include species of the *Lactobacillus*, *Bifidobacterium*, *Streptococcus*, *Enterococcus*, *Bacillus*, *Escherichia*, and *Saccharomyces* genera [35,40].

## 4. *Lactobacillus rhamnosus*

*Lactobacillus rhamnosus* is a rod-shaped, facultative heterofermentative and anaerobic commensal bacterium present in the gastrointestinal tract and maintaining gut homeostasis [60]. It is also Gram-positive, catalase-negative, and non-spore-forming, and it primarily produces lactic acid as its major end product of fermentation. Furthermore, being of the *Lactobacillus genera*, it requires an exogenous nitrogen source such as amino acids or peptides it derives via proteolytic action on casein, the most abundant milk protein [48]. Thus, as a lactic acid bacterium of this genera, proteolysis, lipid hydrolysis, and lactose metabolism are its prime biochemical processes and it is capable of fermenting carbohydrates such as cellobiose, arabinose, and sucrose [61].

*Lactobacillus rhamnosus*, as a probiotic, has been found to function in intestinal flora regulation, antibacterial compound production, and neurotransmitter modulation, with studies reporting its GABA-producing properties that have been found to generate anti-depressant effects and a role in reducing obesity in individuals through probiotic treatment [46,47,48,49].

### Therapeutic Advantages of L. rhamnosus Strains

*L. rhamnosus* and other probiotics in general are safe for human consumption with minimal side effects, except for postoperative, hospitalized, immune-compromised, and critically ill patients in intensive care units. Critically ill infants may experience severe adverse effects such as sepsis, pneumonia, fungemia, GI ischemia, and allergies [10,62]. *L. rhamnosus* strains can also survive pH values below 3.0 and thus have high acid and bile resistance against host defense mechanisms such as gastric activity and bile secretion, enabling them to access the acidic environment of the human gastrointestinal tract (GIT), reach the small intestine, and effectively colonize the host [63].

## 5. *Lactobacillus rhamnosus* Strains and GABAergic Signaling

### 5.1. L. rhamnosus (JB-1)

*Lactobacillus rhamnosus* (JB-1) increases the CNS levels of GABA in mice, modulating GABA_A_ and GABA_B_ receptor expression by activating GABA signaling pathways via vagal afferents [31,32]. Via GABAergic activity, the strain has also been reported to reduce stress-induced plasma glucocorticoid levels in mice, thus averting depression [20,21].

In one study, adult male BALB/c mice administered with the *L. rhamnosus* (JB-1) strain were subjected to fear conditioning tests: the open field sensorimotor test for measuring exploratory behavior, anxiety, and activity and the stress-induced hyperthermia (SIH) test for determining the anxiolytic properties of *L. rhamnosus* (JB-1)-produced GABA [21]. Additionally, the Elevated Plus Maze (EPM) test was used to assess anxiety in CNS disorders, and the Forced Swim Test (FST) was used to estimate the effectiveness of anti-depressants [21]. The results of this study evidenced the positive effects of *L. rhamnosus* (JB-1) on GABA receptor expression, stress-induced corticosterone levels, and depressive behavior. The oral administration of *L. rhamnosus* altered the CNS mRNA expression of GABA_A_ and GABA_B_ receptors while decreasing depressive and anxiety-related behaviors in mice. These higher levels, especially of the GABA_B1b_ receptor mRNA, were found in the cingulate cortex 1 (CG1) and prelimbic (PrL) region cortical areas of *L. rhamnosus* (JB-1)-fed mice with a fully intact vagus nerve in comparison to broth-fed mice [21]. Corticosterone levels induced by stress induction tests were significantly lower in stressed mice that had ingested the *L. rhamnosus* JB-1 strain compared to broth-fed control subjects [20]. With respect to depressive states, the FST analysis revealed that *L. rhamnosus* (JB-1)-fed mice spent significantly more time being active compared to broth-fed mice [21].

In another study [20], adult male BALB/c mice were treated with the *L. rhamnosus* JB-1 strain for 4 weeks and subjected to imaging tests consisting of in vivo magnetic resonance imaging (MRI) and magnetic resonance spectroscopy (MRS) tests, along with an enzyme-linked immunoassay (ELISA) comparison, to measure and validate the concentration changes of cortical metabolites produced due to the treatment [20]. The results of this study also supported findings of orally administered *L. rhamnosus* (JB-1) being able to increase cortical GABAergic activity. In this study, GABA concentrations were found to have significantly increased with JB-1 treatment, as measured in vivo by MRS and ex vivo by ELISA [20]. However, significant elevations (~25%) were only observed 4 weeks into treatment and did not persist after the treatment was stopped [20].

Another study was conducted to measure neurometabolic changes in the hippocampus both before and after *L. rhamnosus* JB-1 ingestion to determine the impact on depressive disorders [4]. In this study, 9-week-old Wistar rats divided into a placebo group and *L. rhamnosus* JB-1 group underwent stress induction using a chronic unpredictable mild stress (CUMS) protocol and were taken through the EPM behavioral test for five weeks; for the next four weeks, each subject from the JB-1 group was fed a fixed quantity of the JB-1 strain (LR-JB1™) daily [4]. Both groups were re-evaluated within those four weeks in the CUMS protocol. In vivo MRI and MRS scans were performed on all subjects from both groups before the commencement of the 4-week feeding period and then right after the end of the entire feeding period [4]. The results from this study showed that after the five weeks of the CUMS and EPM tests, all stressed rats had significantly reduced concentrations of GABA and other metabolites, but after the 4-week period, it was discovered that the LR-JB1 group had their GABA and other metabolites restored to normal levels observed in controls and the placebo group had no restoration of metabolites levels. Additionally, after the four weeks of feeding and testing, the LR-JB1 group demonstrated a more calm and relaxed disposition compared to the placebo group [4].

These studies thus demonstrate the capacity of the *L. rhamnosus* JB-1 strain to improve GABA levels and reduce stress-induced behaviors and depressive states.

### 5.2. L. rhamnosus GG

Another strain, *L. rhamnosus* GG, has been found to increase GABA concentration within fermented adzuki bean milk under optimized cultural conditions [64]. An in vitro study conducted to determine the effects of different commercial lactic acid bacteria on the GABA yield of fermented adzuki bean milk (ABM) showed that *Bifidobacterium bifidum* (BCRC 14615), *Bifidobacterium adolescentis* (BCRC 14606), *Bifidobacterium breve* (BCRC 11846), *Bifidobacterium longum* (BCRC 14634), *Lactobacillus rhamnosus* GG (BCRC 16000), *Lactobacillus acidophilus* (BCRC 14079), *Lactobacillus plantarum* (BCRC 11697), and *Streptococcus salivarius* subsp. thermophilus (BCRC 14085) were the probiotic strains with which the ABM was inoculated and fermented [64]. In each strain’s fermentation group, 1% of the active strain was used and fermentation was carried out for 6 to 60 h, with the response surface methodology (RSM) used to optimize cultural conditions for improving GABA synthesis. After analyses were conducted on each sample’s ABM bacterial count, pH, and GABA levels, the results showed that after 36 h, the inoculation group of *L. rhamnosus* GG had the highest GABA yield among all the groups, 0.44 mg/mL, compared to the initial ABM concentration of approximately 0.05 mg/mL [64]. This study demonstrated the GABA-producing ability of *Lactobacillus rhamnosus* GG and its potential as a probiotic dietary supplement under optimal conditions.

Another study on *L. rhamnosus* GG was conducted on neonatal mouse pups through to their adulthood to determine whether *L. rhamnosus* GG colonization in the early stages of life could reduce anxiety in adulthood [65]. In this study, pregnant mice were fed with *L. rhamnosus* GG until birth, whereupon their newborn offspring were subsequently fed with live or fixed *L. rhamnosus* GG; some were sacrificed, and the rest were weaned, raised in a 12-h reversed light–dark cycle in a temperature-controlled environment, and fed standard rodent chow. Their body weights were measured weekly, and behavioral tests and analyses were conducted on males when they reached adulthood [65]. Fecal tissue samples were collected, after which all mice were sacrificed and all other relevant tissues were collected and analyzed. The results showed increased GABA receptor levels in the hippocampus and amygdala, as well as the relieved anxiety of offspring in adulthood [65].

### 5.3. L. rhamnosus LR-2

Studies on osteoarthritis, a degenerative joint disorder common to the elderly and associated with chronic progressing pain, have also yielded results on the efficacy of the *L. rhamnosus* strain *L. rhamnosus* LR-2 in alleviating pain caused by this illness [66].

Osteoarthritis was first induced in 36.6-week-old male rats, after which they were randomly grouped, with heat-killed *L. rhamnosus* LR-2 fed to some groups every day for 28 days after induction [66]. The rats were then assessed for pain behavior using techniques including nociceptive testing, weight-bearing assessment via incapacitance testing, and histological and immunohistochemical analyses [66]. The results of the study, among other findings, demonstrated a particularly higher expression of GABA within the dorsal root ganglion of rats in the *L. rhamnosus* LR-2-administered group, as well as the corresponding suppression of pain, compared to the control group [66].

### 5.4. L. rhamnosus YS9

In a study directed towards the investigation on *L. rhamnosus* fermentation for GABA production, the culture conditions of the *L. rhamnosus* YS9 strain for high level GABA synthesis were explored [67]. The strain was inoculated within a culture medium and incubated, with pH adjusted every 12 h and a time course analysis carried out on both the culture medium’s extracellular and intracellular GABA contents [67]. The results from the study showed higher extracellular GABA content than intracellular GABA content due to intracellular GABA secretion and extracellular GABA transport. Additionally, optimum glutamate decarboxylase (GAD) activity due to pH adjustments to an optimal value of 4.4 contributed to the highest GABA yield of 187 Mm [67].

### 5.5. L. rhamnosus SP1

A study investigating the beneficial properties of quinoa yogurt beverages used lactic acid strains including those of *Lactobacillus rhamnosus* SP1, *Weissella Confusa* DSM 20194, and *Lactobacillus plantarum* T6B10 for fermentation [68]. Three separate quinoa beverages, B-SP1 from *Lactobacillus rhamnosus* SP1, B-20194 from *Weissella Confusa* DSM 20194, and B-T6B10 from *Lactobacillus plantarum* T6B10 were developed [68]. According to microbiological, chemical, sensory, and nutritional analyses, B-SP1 and B-T6B10 contained the highest GABA levels of up to 211 mg/kg, with *Lactobacillus rhamnosus* SP1 and *Lactobacillus plantarum* T6B10 elevating initial beverage GABA concentrations after inoculation within B-SP1 and B-T6B10, respectively, from 20 to above 100 mg/L [68].

### 5.6. L. rhamnosus HN001

Another study conducted on human subjects [69] revealed the anti-depressant effects of another strain of the *Lactobacillus rhamnosus* bacteria, *Lactobacillus rhamnosus* HN001, in women during pregnancy and 6 months postpartum. This two-year study on postnatal mood involved a double-blind randomized placebo-controlled trial conducted on 423 women at 14–16 weeks of gestation in Auckland and Wellington, New Zealand. The subjects received either a placebo or the HN001 strain on a daily basis from recruitment till the end of the 6-month period post-delivery [69]. Anxio-depressive states were measured using modified versions of the Edinburgh Postnatal Depression Scale (EPDS) and State Trait Anxiety Inventory. The study concluded with a significantly lower prevalence of symptoms of depression and anxiety postpartum in women supplemented with the probiotic HN001 during and after pregnancy compared to those given a placebo [69]. Additionally in the probiotic treatment group, mothers reported significantly lower depression scores than those in the placebo group, even after controlling for factors such as infant colic and time since birth, evidence of the fact that probiotic supplementation remained significantly associated with reduced depression [69]. Though not targeted towards direct measurement of GABAergic activity, this study contributed findings that showed the potential role of *L. rhamnosus* strains in reducing depression in humans.

## 6. Therapeutic Potential of *Lactobacillus rhamnosus*

Strains of *L. rhamnosus* such as JB-1 and HN001 are non-pathogenic bacteria that can regulate depressive states in both humans and mice; particularly, the JB-1 strain can increase cortical GABAergic activity in mice [20,21,69]. Many studies have reported how the inhibition of GABA signals allows for the continuous release of CRF by PVN neurons, ultimately resulting in cortisol overproduction and HPA axis hyperactivity, thus suggesting that such GABAergic activity is a key neurological factor in the maintenance of non-negative, anti-depressive emotional states [9].

Furthermore, the vagus nerve has been implicated as a factor in the successful behavioral and molecular changes induced by *L. rhamnosus* (JB-1) [21], which delineates it as a possible pathway in humans for the functional communication between such microbial strains under study in the gut and the brain that can alter behavioral adaptations toward different stressors [25,70]. These results provide further support for new therapeutic avenues towards remediating depression, anxiety, and chronic negative emotional states to target *L. rhamnosus* vagal afferent GABA as a potential probiotic treatment.

## 7. GABAergic Signaling in Other Probiotic Bacterial Species

In addition *to Lactobacillus rhamnosus* strains, studies have demonstrated the GABA-producing properties of many other bacterial strains from multiple genera (Table 2) [22,65,66,67,69,71,72,73,74].

In an in vitro study investigating the probiotic properties of quinoa yogurt beverages, *Lactobacillus plantarum* T6B10 was found to increase initial GABA concentrations from 20 to above 100 mg/L, with a maximum yield of 211 mg/kg [68]. In an in vivo study investigating the effects of another strain, *L. plantarum* DP189, on treatment in Alzheimer’s disease in mice showed that the strain increased GABA, serotonin, and dopamine levels, thereby reducing neuronal damage and gut dysbiosis [75]. *Lactobacillus reuteri* and *Lactobacillus murine* also restored GABA concentration, caused increases in GABA_A_ receptor alpha-1 subunit (GABRA1) and GABA_A_ receptor alpha-2 subunit (GABRA2) receptor expression, and decreased depressive behavior in Dcf1 knockout mice [76].

Furthermore, in determining the impact of gut microbial GABA on metabolic dysfunction treatment and behavior modification, *Lactobacillus brevis* DPC6108 and *Lactobacillus brevis* DSM32386 were found to lower metabolic dysfunction, improve insulin secretion, and reduce anxiety-related behavior in mouse models of metabolic dysfunction [22]. *Lactobacillus brevis* DPC6108 was again found to significantly increase GABA concentration in a monosodium glutamate (MSG)-containing medium by converting up to 100% of 10 and 20 mg/L MSG to GABA at physiological pH and temperature, with percentages decreasing with increasing concentrations from 30 to 50 mg/L [71]. The *Lactobacillus brevis* TD10 strain isolated from the human gastrointestinal tract was found to have a significantly high GABA-producing potential, synthesizing 5960.82 mg/L of GABA in vitro. The study concluded that under optimized conditions, GABA production could be elevated to 19,960 mg/L [77].

*Bifidobacterium dentium* NCFB2243, *Bifidobacterium dentium* DPC6333, *Bifidobacterium infantis* UCC35624, and *Bifidobacterium adolescentis* DPC6044 (all human-derived strains) were also identified to be GABA-producing microbes. They converted between 22% and 60.9% of 10 mg/L MSG to GABA and between 15.86% and 61.6% of 20 mg/L MSG to GABA, but they decreased in efficiency at increasing concentrations from 30 to 50 mg/L [71]. *Bifidobacterium adolescentis* HD17T2H and *Bifidobacterium adolescentis* PRL2019 were found to have increased expression of glutamic acid decarboxylase-B (gadB) or glutamic acid decarboxylase-C (gadC), which encode glutamate decarboxylase to synthesize GABA, as well as elevated GABA levels, in healthy rat models [72].

To investigate the use of *Streptococcus thermophilus* strains for the production of GABA-enriched fermented milk, *Streptococcus thermophilus* was found to produce GABA in concentrations of up to 2.8 g/L within 48 h [73]. However, when cocultured with *L. rhamnosus* strains, GABA concentrations were increased to 8.3 g/L within an equal 48-h period. This was a highly significant enhancement over the 0.1 g/L GABA concentration produced by individual Lactobacillus strains also cultured and observed [73]. *Streptococcus thermophilus* strains were also reported in previous study to reach GABA-producing concentrations of up to 80 mg/kg, the highest amount of GABA synthesized among the study’s 97 dominant bacterial clusters developed during the maturation of Nostrano cheeses [74].

## 8. Challenges and Future Perspectives

Though the anti-depressant effects of *L. rhamnosus* JB-1 have been demonstrated in mice, studies have shown that these psychotropic benefits do not precisely translate to human populations [78]. This was the result reported from an 8-week randomized placebo-controlled study conducted to determine the impact of *L. rhamnosus* on stress-related behaviors, brain activity, and inflammatory response, as well as the physiology and cognitive performance, of twenty-nine healthy male volunteers aged between 18 and 40 years [78]. In this study, participants each received either one active *L. rhamnosus* capsule or placebo capsule each day and were subjected to cognitive tests from the Cambridge Neuropsychological Test Automated Battery (CANTAB), the socially evaluated cold pressor test (SECPT) acute stress procedure, and EEG testing, along with cortisol and cytokine sampling [78]. The results showed no significant effect of *L. rhamnosus* JB-1 probiotic treatment on acute stress, with no attenuation of cortisol output and self-reports of anxiety and stress levels remaining constant. Additionally, the probiotic did not have any significant impacts on cognitive performance or anti-inflammation compared to the placebo [78]. These findings, conclusive on the absence of psychobiotic effects on healthy individuals, indicate the challenge of *L. rhamnosus* JB-1 application in healthy subjects and expose the need to further investigate the potential of *L. rhamnosus* JB-1 in human subjects afflicted with stress-related disorders including anxiety and depression [78].

An earlier study provided examples of the probiotic specificity of strains and their effect of mismatch among other strains of the same bacterial species. *L. rhamnosus* NCC4007 [79] (a probiotic for anti-obesity and weight control applications) together with *Bifidobacterium longum* NCC3001 were used to investigate whether chronic gut inflammation induces anxiety in mice [79]. In this study, anxiety-like behavior and chronic gut inflammation were induced in mice through the administration of the *T. muris* parasite. The infected mice were then treated with the *B. longum* and *L. rhamnosus* strains, and the results from inflammation assessments and behavioral tests showed the normalization of hippocampal brain-derived neurotropic factor (BDNF) levels and decreased anxiety and altered behavior, respectively, during treatment with *B. longum* NCC3001 [79]. No significant improvement in either anxiety relief or gastrointestinal inflammation was observed during treatment with the *L. rhamnosus* NCC4007 strain [79].

Though many strains of *Lactobacillus rhamnosus* exhibit GABA-ergic properties and anti-depressant effects in subjects, these properties have been found to not cross over to all bacterial strains or from animal to human models [78,79].

In view of this, more metagenomic studies on human-derived bacterial strains of *L. rhamnosus* should be conducted to screen for other possible genetic factors associated with GABA production, as it has been reported that only a fraction of bacterial strains with glutamic acid decarboxylase-B (gadB) or glutamic acid decarboxylase-C (gadC) genes are capable of GABA production [22,73]. Additionally, more human studies should be conducted exclusively on individuals suffering from depressive or anxiety-related disorders to accurately gauge the impact of GABA-producing strains in animal models, which could be due to possible disparities between microbial strain impact on healthy individuals and compromised individuals [78].

On a broader scale, more studies can be geared towards investigating the probiotic dynamics underlying the interactions between co-cultured microbial strains such as those reported on the enhanced GABA yield of co-cultured *Lactobacillus rhamnosus* and *Streptococcus thermophilus* strains, as these could pave the way for novel multi-strain therapies that are more potent than those of their individual constituents strains, in a manner such that the whole does indeed become greater than the sum of its parts [73].

## 9. Conclusions

In this review, the GABAergic activity and potential of *Lactobacillus rhamnosus* strains were discussed as plausible therapeutic avenues for attenuating the depression–inducing HPA axis hyperactivity in addiction withdrawal. Multiple avenues have also been recommended for the further investigation of various criteria such as strain genetic factors, physiological conditions, or microbial population levels and interactions in the gastrointestinal tract that enable or prevent GABA production by the *Lactobacillus rhamnosus* strains of the human gut microbiota.

## Figures and Tables

**Table 1:** Common bacterial genera in probiotics applications including their physiological functions and their areas of therapeutic uses.

Genera	Species	Physiological Function	Therapeutic Agency	References
*Lactobacillus*	*Lactobacillus rhamnosus*	Intestinal flora regulation Antibacterial compound production Neurotransmitter modulation Immune response regulation	Depression	[46,47,48,49]
			Obesity	
	
	*Lactobacillus reuteri*	Immune response regulation Intestinal barrier function Intestinal, oral and vaginal flora regulation	Obesity Gastrointestinal disorders Acute infectious diarrhea	[47,48]
*Bifidobacterium*	*Bifidobacterium longum*	Immune response regulation Carbohydrate metabolism in infants Lactose metabolism	Acute rotavirus Diarrhea Antibiotic-associated diarrhea Lactose intolerance	[50,51]
	*Bifidobacterium* *breve*	Pathogen growt hinhibition Immune response regulation Intestinal flora regulation Lipid metabolism	Celiac disease Obesity Necrotizing enterocolitis	[50,52]
*Streptococcus*	*Streptococcus thermophilus*	Immune response regulation Lactose metabolism Intestinal toxin regulation Immune response regulation	Lactose intolerance Uremia	[53,54]
*Bacillus*	*Bacillus coagulans*	Lactose metabolism Protein and carbohydrate metabolism Toxin regulation Bowel motility Cholesterol metabolism Intestinal flora regulation	Irritable bowel syndrome Antibiotic-associated diarrhea Obesity Inflammatory bowel disease Colorectal cancer	[47,55]
*Saccharomyces*	*Saccharomyces cerevisiae*	Immune response regulation Pathogen growth inhibition	Inflammatory bowel disease Anti-biotic associated diarrhea Vaginal infection	[47,56]
*Enterococcus*	*Enterococcus faecium*	Cholesterol modulation Immune response regulation Intestinal flora regulation	Intestinal disease Anti-biotic associated diarrhea Irritable bowel syndrome	[56,57]
*Escherichia*	*Escherichia coli*	Intestinal barrier Immune response regulation	Intestinal inflammatory diseaseInfectious diarrhea	[58,59]

**Table 2:** Common GABA-producing bacterial species, strains, and their effects.

Genera	GABA-Producing Species/Strains	Probiotic GABAergic Effects/Correlations	References
*Lactobacillus*	*Lactobacillus*	↓ Depression	[4,20,21]
	*rhamnosus* JB-1		
	*Lactobacillus*	↓ Anxiety-like	[64,65]
	*rhamnosus* GG	Behavior	
	*Lactobacillus*	↓ Osteoarthritis pain	[66]
	*rhamnosus* LR-2	Severity	
	*Lactobacillus*	N/A (In vitro)	[67]
	*rhamnosus* YS9		
	*Lactobacillus rhamnosus* SP1	N/A (In vitro)	[68]
	*Lactobacillus plantarum* T6B10	N/A (In vitro)	[68]
	*L. plantarum* DP189	↓ Gut dysbiosis,	
		↓ Cognitive dysfunction in Alzheimer’s disease	[75]
	*Lactobacillus reuteri*	↓ Depression	[76]
	*Lactobacillus murine*	↓ Depression	[76]
	*Lactobacillus*	↑ Insulin secretion,	
	*brevis* DPC6108	↓ Metabolic	[22,71]
		Dysfunction,	
		↓ Depression-like behavior	
	*Lactobacillus*	↑ Insulin secretion,	[22,71]
	*brevis* DSM32386	↓ Metabolic	
		Dysfunction,	
	*Lactobacillus*	↓ Depression-like behavior	[77]
	*brevis* TD10	N/A (in vitro)	
*Bifidobacterium*	*Bifidobacterium*	N/A (in vitro)	[71]
	*dentium* NCFB 2243		
	*Bifidobacterium*	N/A (in vitro)	[71]
	*dentium* DPC6333		
	*Bifidobacterium infantis* UCC35624	N/A (in vitro)	[71]
	*Bifidobacterium*	N/A	[72]
	*adolescentis* PRL2019		
	*Bifidobacterium adolescentis* HD17T2H	N/A	[72]
	*Bifidobacterium adolescentis* DPC6044	N/A (in vitro)	[71]
*Streptococcus*	*Streptococcus thermophilus* GABA	N/A (in vitro)	[73,74]

N/A denotes not applicable; ↓ and ↑ denote decreasing and increasing levels, respectively.

## Data Availability

Not applicable.
